# Improving the Hydrophobicity of Plasticized Polyvinyl Chloride for Use in an Endotracheal Tube

**DOI:** 10.3390/ma16227089

**Published:** 2023-11-08

**Authors:** Lavinia Marcut, Aurel George Mohan, Iuliana Corneschi, Elena Grosu, Gheorghe Paltanea, Ionela Avram, Alexandra Valentina Badaluta, Gabriel Vasilievici, Cristian-Andi Nicolae, Lia Mara Ditu

**Affiliations:** 1 Faculty of Medicine and Pharmacy, University of Oradea, 10 P-ta 1 December Street, RO-410073 Oradea, Romania; rat_lavinia@yahoo.com (L.M.); mohanaurel@yahoo.com (A.G.M.); 2 Intensive Care Unit, Clinical Emergency Hospital Oradea, 65 Gheorghe Doja Street, RO-410169 Oradea, Romania; 3 Department of Neurosurgery, Clinical Emergency Hospital Oradea, 65 Gheorghe Doja Street, RO-410169 Oradea, Romania; 4 Faculty of Material Science and Engineering, National University of Science and Technology Politehnica Bucharest, 313 Splaiul Independentei, District 6, RO-060042 Bucharest, Romania; grosu@yahoo.com; 5 Faculty of Electrical Engineering, National University of Science and Technology Politehnica Bucharest, 313 Splaiul Independentei, District 6, RO-060042 Bucharest, Romania; gheorghe.paltanea@upb.ro; 6 Faculty of Biology, Botanic and Microbiology Department, University of Bucharest, 3, Aleea Portocalelor, District 5, Grădina Botanică, RO-050095 Bucharest, Romania; ionela.avram@bio.unibuc.ro (I.A.); badaluta.valentina@s.bio.unibuc.ro (A.V.B.); lia_mara_d@yahoo.com (L.M.D.); 7 National Institute for Research & Development in Chemistry and Petrochemistry ICECHIM, 202 Splaiul Independenței, District 6, RO-060021 Bucharest, Romania; gvasilievici@icechim.ro (G.V.); cristian.nicolae@icechim.ro (C.-A.N.)

**Keywords:** endotracheal tube, plasticized PVC in medical devices, attachment of microorganisms on PVC, increasing the hydrophobicity of PVC, plasma treatment in SF6 discharge, magnetron sputtering to coat PVC with PTFE

## Abstract

An endotracheal tube (ETT) is a greatly appreciated medical device at the global level with widespread application in the treatment of respiratory diseases, such as bronchitis and asthma, and in general anesthesia, to provide narcotic gases. Since an important quantitative request for cuffed ETTs was recorded during the COVID-19 pandemic, concerns about infection have risen. The plasticized polyvinyl chloride (PVC) material used to manufacture ETTs favors the attachment of microorganisms from the human biological environment and the migration of plasticizer from the polymer that feeds the microorganisms and promotes the growth of biofilms. This leads to developing infections, which means additional suffering, discomfort for patients, and increased hospital costs. In this work, we propose to modify the surfaces of some samples taken from commercial ETTs in order to develop their hydrophobic character using surface fluorination by a plasma treatment in SF6 discharge and magnetron sputtering physical evaporation from the PTFE target. Samples with surfaces thus modified were subsequently tested using XPS, ATR-FTIR, CA, SEM + EDAX, profilometry, density, Shore A hardness, TGA-DSC, and biological antimicrobial and biocompatibility properties. The obtained results demonstrate a successful increase in the hydrophobic character of the plasticized PVC samples and biocompatibility properties.

## 1. Introduction

It is important to acknowledge that, in hospitals’ intensive care units (ICUs), indwelling devices, such as catheters and feeding tubes, can create a pathway for both exogenous and endogenous microorganisms to circulate, thus exposing the human body to external media. This work aims to improve the surface quality of endotracheal-tube (ETT) catheter-type medical devices by increasing their hydrophobic character.

A constant concern of those in this field is the knowledge of the biological response that biomaterials induce in human tissues, blood, or other biological fluids. The biological response may include changes in cell structure, metabolic state, tissue structure, and function. Regarding the applications of biomaterials in urinary implants, in addition to the efficiency of the medical act, the interaction between the polymeric material and the human body is of interest. The external surface of the medical devices must be smooth, without deformations, cracks, or roughness, in order not to cause damage to internal organs. The appropriate use of the catheter can be determined by the use period in the human body. Medical devices can provide conditions for bacterial colonization because a biofilm is produced on their surface. These biofilms accumulate crystalline mineral deposits, minimizing the effect of antibiotic agents.

At present, most tubular medical devices on the market are based on plasticized PVC. Compared to silicone rubber, plasticized PVC allows technological operations in the industrial manufacturing of medical devices, such as the atraumatic thermal closure of one end of the tube and solvent polishing, as well as the polishing, cord closure, threading, flaring, stiffening, bending, and assembly with connectors and other tubing by adhesive bonding. For these reasons, plasticized PVC can be used to make all types of tubular medical devices. On the other hand, silicone rubber can only be used to make perforated or non-perforated drains. Medical-grade polyvinyl chloride (PVC) is the ideal choice for the manufacture of medical devices due to its numerous advantages, such as versatility, flexibility, good processing in a molten state, high transparency, resistance to corrosion and chemical substances, durability, biocompatibility, low cost, and the ability to be extruded into various sizes, shapes, and colors. Additionally, medical PVC tubing can feature one or more lumens and can be compounded to offer different flexibilities. It supports repeated sterilization and can achieve smooth inner and outer surfaces that provide non-irritating contact with the human body and allow for a non-turbulent flow of fluids through drains or catheters. However, the use of PVC in the manufacture of medical devices is conditioned by compounding with different chemical substances to offer different flexibilities. Among these, the most important are plasticizer (commonly Di(2-ethylhexyl) phthalate or DEHP), stabilizer (such as Stanclaire), epoxidized soybean oil (ESBO), and other processing aids. For the long-term use of drains or catheters in contact with different human environments in the lungs, bladder, kidneys, nasal or buccal cavities, trachea, esophagus, stomach or biological fluids, such as blood, saliva, or other secretions, it is critical to consider the diffusion of plasticizers through the PVC material and their removal from the polymeric composition. In addition, the plasticizers can transport unwanted elements, such as unreacted monomers, catalyst traces, processing modifiers, and degradation products.

For these reasons, the plasticizer migration phenomenon must be considered and controlled when making PVC mixtures. The time it takes for substances to migrate from the polyvinyl polymer compounds depends on the following factors: temperature, the concentration of components with a low molecular mass, contact surface condition, and the nature of the contact medium.

Last but not least, the polar nature of PVC must be taken into account due to the chlorine content combined with the hydrophilic character of the material, which leads to favoring the attachment of Gram-positive and Gram-negative microorganisms and fungi found in different parts of the human body. This is particularly significant in digestive, respiratory, or urinary tract devices. Several medical devices can be inserted into the human body, including the nasogastric tube, tracheostomy tube, endotracheal catheter, and urinary catheter. These devices should be utilized for a maximum of 72 h due to the potential for microorganisms to become attached and produce biofilms. For this reason, considerable research has focused on modifying the wettability of the inner or outer surface of the tube to prevent the attachment of microorganisms and the formation of biofilms. Factors influencing biofilm formation include pH levels, humidity, surface wettability, temperature, and surface polarity. These biofilms can become substantially thick, in the range of 45–100 µm [1,2], because microorganisms can grow by consuming additives that diffuse from PVC into human media [3,4,5].

Considerable research has been conducted to alter the surfaces of plasticized PVC medical devices to reduce or prevent the attachment of microorganisms and the formation of biofilms [6,7]. The two types of techniques that have been investigated include surface coating with ceragenin-type peptides and plasma treatment [8,9,10]. An additional approach that has been used is to develop antibacterial and antifungal ceragenins as non-peptides [11,12,13,14,15].

Ceragenins act as antibacterial agents against Gram-positive and Gram-negative bacteria, such as Staphylococcus aureus, Acinetobacter baumannii, Pseudomonas aeruginosa, Streptococcus mutans, and Lactobacillus casei, as well as against strains found in oral infections, Staphylococcus aureus ATCC 29213, Streptococcus salivarius ATCC 13419, Streptococcus sanguinis ATCC 10556, Streptococcus mutants ATCC 35668, Enterococcus faecalis ATCC 29212, Moraxella catarrhalis ATCC 23246, Peptostreptococcus anaerobius ATCC 27337, Lactobacillus casei ATCC 393, and Fusobacterium nucleatum ATCC 25586 [16,17,18]. Ceragenins mimic the activity of cationic antimicrobial peptides (CAMPs) and are capable of killing bacteria using a membrane depolarization mechanism, especially in Gram-positive bacteria [19]. In Gram-negative bacteria, the mechanism seems to be different, as caragenins induce the dysregulation of the phosphate-dependent transporting mechanisms in the inner membrane [20]. The fungicidal properties of ceragenins were tested against Candida spp., Cryptococcus, Aspergillus, Scedosporium, Rhizopus, and Blastomyces [21]. Microscopy studies show that treatment with ceragenins causes Candida cells to undergo extensive surface changes, indicating surface membrane damage, similar to natural AMPs [22]. The significant antibiofilm activity of ceragenins was observed when researchers successfully inhibited the biofilm formation of Pseudomonas aeruginosa phenotypes by penetrating the biofilm within 30 min and strongly associating it with anionic cell surfaces, which induce the formation of transient pores in the membrane, resulting in membrane depolarization and cell death [23,24]. Additionally, ceragenins have been shown to possess sporicidal activity, for example, against Bacillus subtilis, in both vegetative and spore forms [25]. Ceragenins exhibit antiviral effects against Herpes simplexvirus [26] and Vacciniavirus [27], and antiparasitic activity against Paramecium caudatum [28], Caernohabditis elegans [29], Trypanosoma cruzi, Leishmania major, Acanthamoeba castellanii [30], Bacteroides fragilis, Propionibacterium acnes, Clostridium difficile, Bacteroides thetaiotaomicron, Bacteroides stercoris, Provotella melaninogenica, Prevotella oralis, Prevotellabivia, Provotella disiens, Clostridium perfrigens, and *Peptostreptococcus* spp. [31,32].

Carrageenan, which has useful antimicrobial and antifungal properties, was tested as a coating inside and outside an endotracheal tube [33]. This was conducted by immersing the tube in a hydromer-coating solution, 2018-20M. The resulting layers showed effective antimicrobial and biofilm growth-inhibiting properties.

Previous studies have explored the ways to enhance PVC by applying a polytetrafluoroethylene (PTFE) layer through vacuum thermal evaporation and treating it with carbon tetrafluoride (CF_4_). This method has successfully produced a highly hydrophobic surface with a contact angle of 150°, as reported in [23,34]. In addition, several other studies have investigated the effects of coating silicon wafers that possess smooth surfaces or carbon nanowall (CNW)-deposited layers [35,36,37,38,39]. Two methods have been utilized to cover the surfaces with PTFE: the deposition of PTFE-like layers using magnetron sputtering and surface fluorination via the plasma-assisted chemical vapor deposition of tetrafluoroethane (C_2_H_2_F_4_) or plasma treatment in sulfur hexafluoride (SF_6_) discharge. The results showed that the surfaces exhibited significant improvement in hydrophobicity [40,41] and antimicrobial properties [42].

Based on these findings, we employ the PTFE deposition methods mentioned earlier [43,44] to address the issue of microorganisms adhering to endotracheal tube surfaces. Through these methods, we successfully coated the plasticized PVC surfaces with a layer of PTFE, resulting in an increased contact angle as well as hydrophobic properties. Additionally, our biological studies revealed enhanced biocompatibility and antimicrobial properties.

In this paper, we investigate the existence of the fluorine (F) and the PTFE coating through X-ray photoelectron spectroscopy (XPS), perform a surface analysis based on scanning electron microscopy (SEM), monitor the possible changes in the contact angle (CA) of the PVC after the two applied treatments, and perform roughness determination for the three types of samples. Then, we conducted a thermal analysis through thermogravimetric analysis (TGA) and differential scanning calorimetry (DSC), investigate the sample density and mechanical properties, such as Shore A hardness, and perform antimicrobial and cytotoxicity analyses.

## 2. Materials and Methods

### 2.1. Materials

#### 2.1.1. General Presentation of the Materials Used

**Figure 1:**
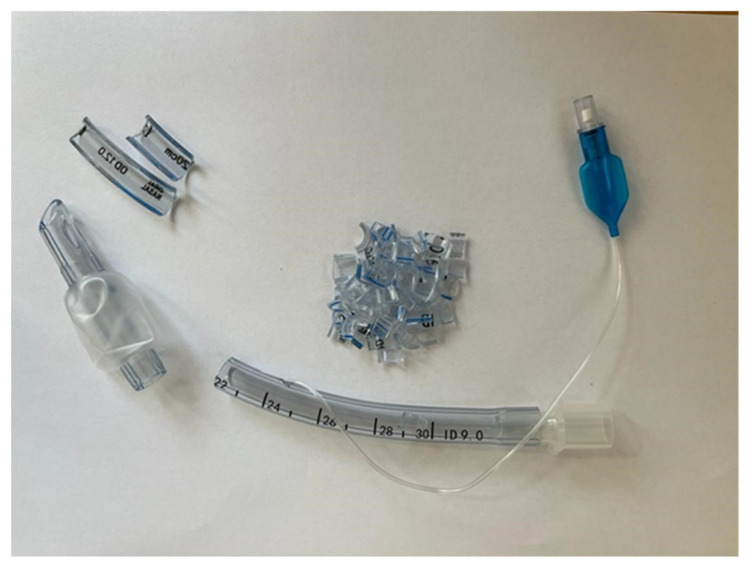
Samples from the endotracheal tube.

To determine the efficiency and influence of two different surface treatments on the endotracheal tubes, we used plane samples collected from plasticized polyvinyl chloride (PVC) endotracheal catheters produced by Nanchang Kaimed Medical Apparatus Co., Ltd. from Nanchang City, Jiangxi Province, China [45], under the Medical Device Directive 93/42/EEC [46]. We took samples from the material of the PVC endotracheal tube (Figure 1).

Due to the fact that the tubes have a curved surface, we proceeded to flatten them by applying a heat treatment as indicated by Bormashenko et al. [47]. To avoid the degradation of the PVC material, we decided to expose the samples only to a heating procedure until an overall softening of the material occurred, and then we pressed the samples. More precisely, we inserted the PVC samples between the platens of a Brabender-type press and subjected it to softening at 100 °C, a temperature that is higher than 70 °C (the softening point of PVC); pressing at a pressure of 500 bar for 10 min; and then cooling to 25 °C to produce samples with a flat surface. After that, the samples were subjected to surface treatment and analyses. The polytetrafluoroethylene sputtering target (MSE PRO Teflon (PTFE), 99.9% purity) was purchased from MSE Supplies, Tucson, AZ, USA, and had a diameter of 50.8 mm and a thickness of 3.175 mm.

#### 2.1.2. PVC Sample Surface Treatment

##### Surface Fluorination by Plasma Treatment in SF6 Discharge

A spherical stainless-steel chamber, in which vacuum was generated with a turbomolecular/rotary pumping system, was used to perform the surface fluorination of the sample in the SF6 plasma discharge medium. A plasma-activated chemical vapor deposition (PCVD) source was mounted at a distance of 9 cm and an angle of 45° to the grounded electrode that consisted of a rotating substrate holder. SF6 gas was introduced into the chamber mentioned above at a constant flow rate of 25 sccm/min in order to increase the working pressure from 2.3 × 10^−5^ mbar to 1.9 × 10^−4^ mbar. The treatment time was set at 5 min, and it was performed at a radio frequency (RF) source of 40 W active power. Then, the RF source was shut down, and the samples were left inside for another 30 min under the action of SF6 gas.

##### Magnetron Sputtering Physical Evaporation from the PTFE Target

The second surface treatment applied on the PVC samples consisted of a deposition process of fluorinated layers from a solid polytetrafluoroethylene (PTFE) target. The same chamber presented in the first treatment was involved, but in this case, it was equipped with a magnetron head (Kurt J. Lesker, Dresden, Germany) placed exactly in the same fashion as the PCVD source. A Pfeiffer baratron CCR 375, 0.1 Torr F.S., DN 16 ISO KF (Pfeiffer Vacuum, Bucharest, Romania) vacuum gauge monitored the room pressure, which was kept constant at 2.3 × 10^−5^ mbar, while the SF6 flow rate of 25 sccm/min and argon flow rate of 100 sccm/min were controlled by electronic mass flow controllers (Bronkhorst, AK Ruurlo, Ruurlo, The Netherlands). The second treatment was applied for 10 min in the gas-combined atmosphere, and then the sputtering deposition of PTFE coating was performed for 5 min at 80 W active power of the RF source. The thickness of the coating was about 20 nm.

The samples were coded as follows: ED0: control samples made of the pristine and untreated material; ED1: samples subjected to surface fluorination by plasma treatment in SF6 discharge; and ED2: samples subjected to magnetron sputtering physical evaporation from the PTFE target.

### 2.2. Characterization Methods

#### 2.2.1. The Chemical Structures of Samples ED0, ED1, and ED2

##### X-ray Photoelectron Spectroscopy (XPS)

The chemical composition of the samples was assessed based on X-ray photoelectron spectroscopy (XPS). These investigations were performed with a K-Alpha Thermo Scientific (Waltham, MA, USA) spectrometer that contains a hemispherical analyzer. An aluminum anode (AlKα, 1486.6 eV) was irradiated with X-rays and generated excitation photoelectrons at an emission current of 3 mA and a tube voltage of 12 kV. The standard C1s peak (284.6 eV) was used to calibrate the peak position, and high-resolution spectra for F1s, C1s, N1s, and O1s were determined to find the chemical bonding in the material.

##### Attenuated Total Reflection Fourier-Infrared (ATR-FTIR) Spectroscopy

The analyses were performed using an ATR-FTIR Jasco 6200 apparatus (Jasco Inc., Easton, MD, USA). For each sample, 60 scans at a resolution of 4 cm^−1^ were conducted.

#### 2.2.2. Surface Analysis

##### Scanning Electron Microscopy (SEM) and Energy Dispersive X-ray Analysis (EDAX)

To study the morphology of the samples’ material, we used a Philips XL 30 ESEM TMP Scanning Electron Microscope (FEI Company, Eindhoven, The Netherlands). The polymeric samples denoted as ED0, ED1, and ED2 were prepared without any previous preparation.

##### Contact Angle Measurements

The ETT samples’ wettability was determined using the contact angle method. We used a Krüss Drop Shape Analyzer, DSA-1000 (A. Krüss Optronic GmbH, Hamburg, Germany), which allows contact angle measurements with three liquids, such as water (W), diiodomethane (DIM), and ethylene glycol (EG). The sample investigations were conducted at a room temperature of 23 ± 5 °C in a medium with a humidity of 45 ± 5%. We performed an average of three determinations per wetting agent and sample. The surface free energy (SFE) was computed based on the Owens, Wendt, Rabel, and Kaelbe (OWKR) method [36]. To determine the wetting characteristics of the surface, different volumes of liquid were used, approximately 1–15 microliters, depending on the surface available on the sample and, at the same time, to eliminate the errors that can be induced by the volume variation of the drop. The dosing of the drops was conducted with the automatic dosing system of the tensiometer and with the help of micrometric syringes. The figures show aspects of the drops with larger volumes (10–15 microliters).

##### Profilometry Analysis

The device used to investigate the surface roughness was a Form Talysurf^®^ i-Series PRO Range (Taylor Hobson Ametek, Warrenville, IL, USA) equipped with a transducer with a standard probe for measuring flat surfaces and using Metrology 4.0 Software (Taylor Hobson Ametek, Warrenville, IL, USA). The surface roughness was determined according to standars in force [48]. The following settings were chosen: an applied contact force of <1 mN at a scan speed of 1 mm/s for a 10 mm scan length. The parameters Ra (arithmetic average deviation from the mean line) and Rq (root-mean-square average of the profile heights over the evaluation length) were determined for each sample based on five observations.

#### 2.2.3. Thermal Analysis

The thermal stability of samples was investigated using a Hi-Res TGA Q5000 V3.13 (TA Instruments, New Castle, DE, USA) by analyzing the thermogravimetric (TGA) behavior of the samples in duplicate, between 25 °C and 750 °C, at a heating rate of 10 °C/min. The samples ED0, ED1, and ED2 with masses of 11.4–11.6 mg were heated in a 100 µL platinum pan. The purge gases were nitrogen with a flow rate of 50 mL/min and synthetic air with a flow rate of 50 mL/min. The maximum degradation characteristic was determined from the TGA curves as the weight loss.

DSC analyses were performed on a DSC Q2000 (TA Instruments, New Castle, DE, USA). The samples ED0, ED1, and ED2 were heated in a 100 µL Tzero Aluminum pan. The purge gas was helium at a flow rate of 25 mL/min from −60 °C to 750 °C and the heating rate was 10 °C/min. The samples were cooled to –75 °C, equilibrated for 2 min, heated to 200 °C at a heating rate of 10 °C/min (first heating), equilibrated for 2 min, cooled to −75 °C at 10 °C/min (cooling cycle), equilibrated for 2 min, and heated again to 200 °C at 10 °C/min (second heating). In the cooling cycle, the crystallization temperature (Tc) was recorded from the crystallization exotherm as the peak temperature. In the second heating, the melting temperature (Tm) was recorded from the melting exotherm as the peak temperature.

#### 2.2.4. Material Density Measurement and Hardness Shore A Analyses

The sample density was determined based on an analytical balance with four decimals (Radwag AS 220/R2, Warsaw, Poland). The measurements were performed according to the ISO 1183-1:2013 standard, as presented in [49].

The hardness Shore A was measured using a DD-300 Digital Precision Durometer (Checkline, Birmingham, UK), and the testing method followed ISO 868:2003 [50].

#### 2.2.5. Antimicrobial and Cytotoxicity Analyses

##### Antimicrobial Tests

The quantitative evaluation of the anti-adherence and antibiofilm activity of the three tested samples was conducted by the quantification of the bacterial cells adhered to the surface of the samples after 24 h of incubation (adherend cells) and of the bacterial cells included in the biofilm developed on the surface of the tested samples after 72 h of incubation. For this purpose, sterile plates with 24 wells were filled with 1 mL of the liquid culture medium (Muller–Hinton (MH) agar for bacterial strains bought from Condalab, Madrid, Spain, and Sabouraud agar for the yeast strains supplied by Scharlau Group, Barcelona, Spain), which was inoculated with 10 µL of the standardized bacterial suspension (at a 1.5 × 10^8^ CFU/mL density, corresponding to McFarland 0.5 and in accordance with the CLSI standard method [51]), followed by immersion of the sterile, tested samples. The plates were incubated at 37 °C for 24 and 72 h. At 24 h after incubation, the microbial cells went through a multiplication cycle, and when they reached a critical density, they began to adhere to the surface of the materials. After 72 h of incubation, the monospecific biofilm started to develop on the surface of the materials.

After 24 and 72 h of incubation, each sample was removed from the medium, gently washed three times with phosphate-buffered saline (PBS) to remove non-adherent cells, and placed in sterile centrifuge tubes containing 1 mL of PBS. The samples were mixed vigorously in the shaker for 1 min and sonicated for 10 s. The quantitative evaluation of cell density (both adhered cells and biofilm-included cells) was assessed using the VCC (viable cell count) method.

The VCC method aims to record the actively dividing/growing bacterial cells that adhered to or formed a biofilm, using a series of dilutions, from 10^−1^ to 10^−8^, in PBS (phosphate-buffered saline). Subsequently, 10µL of the appropriately diluted sample was spotted in triplicates, on an MH agar plate, and incubated overnight at 37 °C. After that, the number of colonies formed on the plate were determined for the last dilution at which the colonies were countable. The results were expressed as colony-forming unit/mL (CFU/mL), representing the average of the colony number determined in the three spots/dilutions and multiplied by the set dilution value.

##### Cytotoxicity Analysis

The cytocompatibility of the tested samples was evaluated by measuring the viability of a human colorectal adenocarcinoma cell line (HT-29) using the MTT reagent (Sigma, Santa Clara, CA, USA). HT-29 cells were grown in RPMI 1640 medium (Lonza, Switzerland) supplemented with 10% FBS (fetal bovine serum) and Pen-Strep (Biochrom, Germany) in 24-well polystyrene plates. The cells were incubated at 37 °C in 5% CO_2_ until they reached a confluence of 70%, passage 24. Subsequently, the medium was removed, and the cells were incubated with 1 mL RPMI 1640 medium with 10% FBS, the antibiotic Pen-Strep, and biomaterial samples with 0.25 cm^2^ for 24 h under the same conditions. After incubation, the medium and the biomaterials were removed, and the cells were washed twice with warm PBS and incubated with 100 µg/mL MTT solution for 2.5 h at 37 °C in a 5% CO_2_ atmosphere. After incubation, the dye was solubilized with dimethyl sulfoxide (DMSO), and the plate was read at 540 nm using a Synergy HTX spectrophotometer by Agilent Technology Biotek from Santa Clara, CA, USA. Cell viability was calculated using the following formula [52]:

% survival = (mean experimental absorbance/mean control absorbance) × 100(1)

## 3. Results and Discussion

### 3.1. Chemical Structure Analyses

#### 3.1.1. X-ray Photoelectron Spectroscopy (XPS)

**Figure 2:**
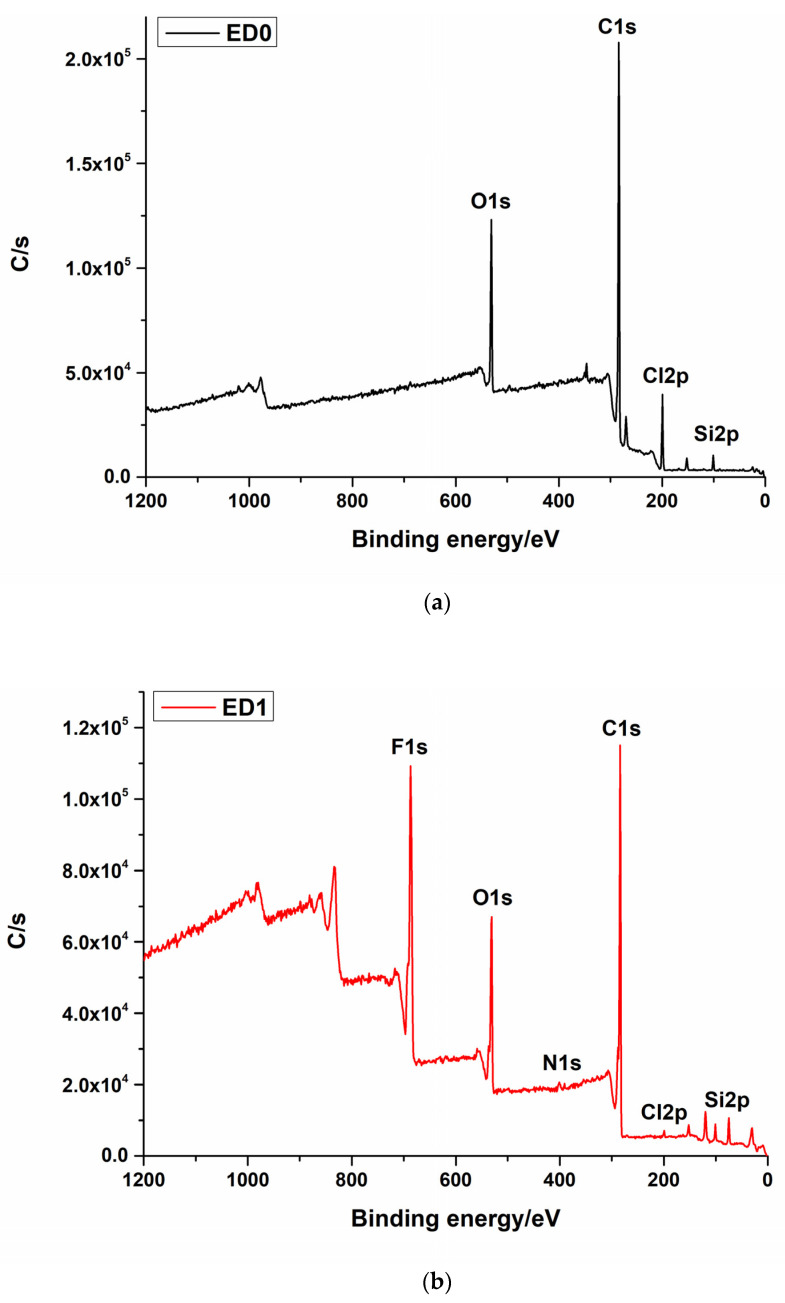

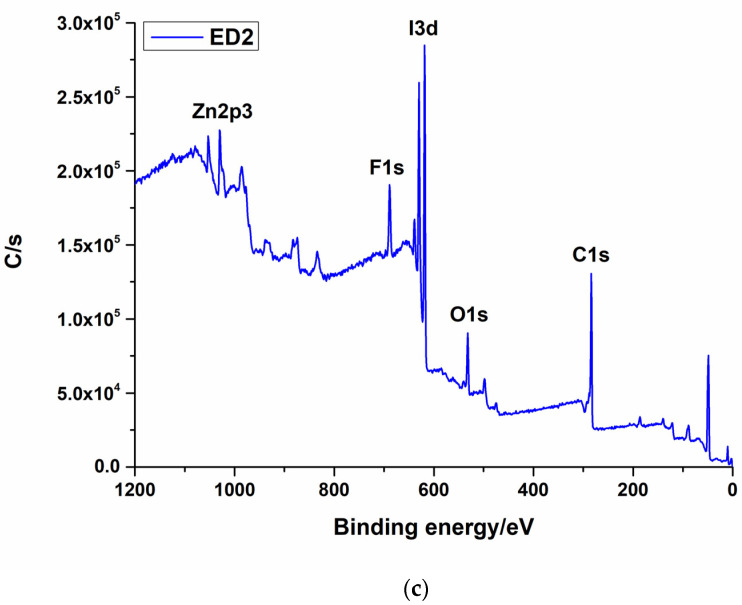
XPS survey spectra for the (**a**) ED0, (**b**) ED1, and (**c**) ED2 samples.

The treatment generated using the SF6 gas led to the formation of an F1s layer at the surface of the catheter. This was proved by the presence of an F1s peak in the XPS spectra [53,54]. The presence of N1s can be explained by the inert gas used during the plasma treatment. The treatment generated by magnetron sputtering from the PTFE target also induced the deposition of the fluorine layer, but in a small quantity, because, in this case, fluorine is generated from a fluor-containing solid material by a sputtering process (Figure 2a–c). This fact also explains the presence of I3d and Zn2p3 in the chamber material [35,55].

Table 1 shows the atomic composition of the experimental samples.

#### 3.1.2. Attenuated Total Reflection Fourier-Infrared (ATR-FTIR) Spectroscopy

**Figure 3:**
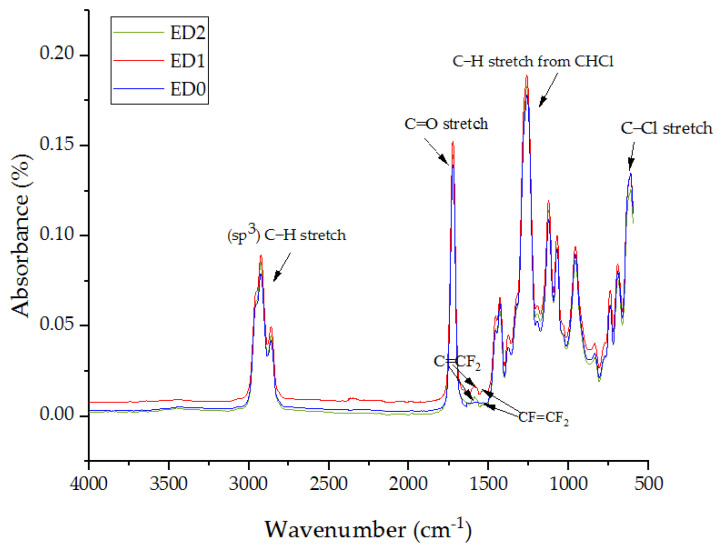
FTIR analyses for the samples ED0, ED1, and ED2.

Figure 3 exhibits the FTIR spectra for the samples ED0, ED1, and ED2. We can observe that there are no major differences between the absorption bands of the analyzed samples. All samples—ED0, ED1, and ED2—present the same intense IR bands, as follows: at 2925 cm^−1^, the stretch band was attributed to C-H bonds; at 1725 cm^−1^, the stretch band was attributed to the peroxide group C = O; at 1264 cm^−1^, the extended band with the highest absorbance was attributed to C-H hydrogen bonds belonging to the CHCl functional group; and at 614 cm^−1^, the stretch band corresponded to the C-Cl functional group. In addition, the samples ED1 and ED2 presented two intense bands at 1588 cm^−1^ and 1535 cm^−1^, attributed to the asymmetric bonds C = CF_2_ and CF = CF_2_, respectively [56]. The copolymer of tetrafluoroethylene (PTFE) is known for its helical structure [37,57], associated with the left-handed and right-handed character, which denotes the partial crystalline component in the case of the initial target [38]. The IR spectrum of plasticized PVC overlaps with the IR spectrum of PTFE, and both show several additional peaks in the range of 1500 cm^−1^–500 cm^−1^, confirming a complex structure with numerous C = CF_2_ and CF = CF_2_ bonds and functional groups contributing CF_3_ [37,58] and CF [39]. In addition, we know from other scientific research that PTFE can present the main absorption peaks at 1800 cm^−1^, 1213 cm^−1^ (or 1230 cm^−1^ or 1200 cm^−1^), and 1155 cm^−1^ (or 1146 cm−^1^) attributed to CF_2_ symmetric and asymmetric bands [59]. These peaks are not observable in the graph in the figure as they coincide with the corresponding peaks of the plasticized PVC polymer. We also know from other articles that PTFE can present absorption peaks in the cm^−1^ range [37,57,60,61,62].

### 3.2. Surface Analysis

#### 3.2.1. Scanning Electron Microscopy (SEM)

**Figure 4:**
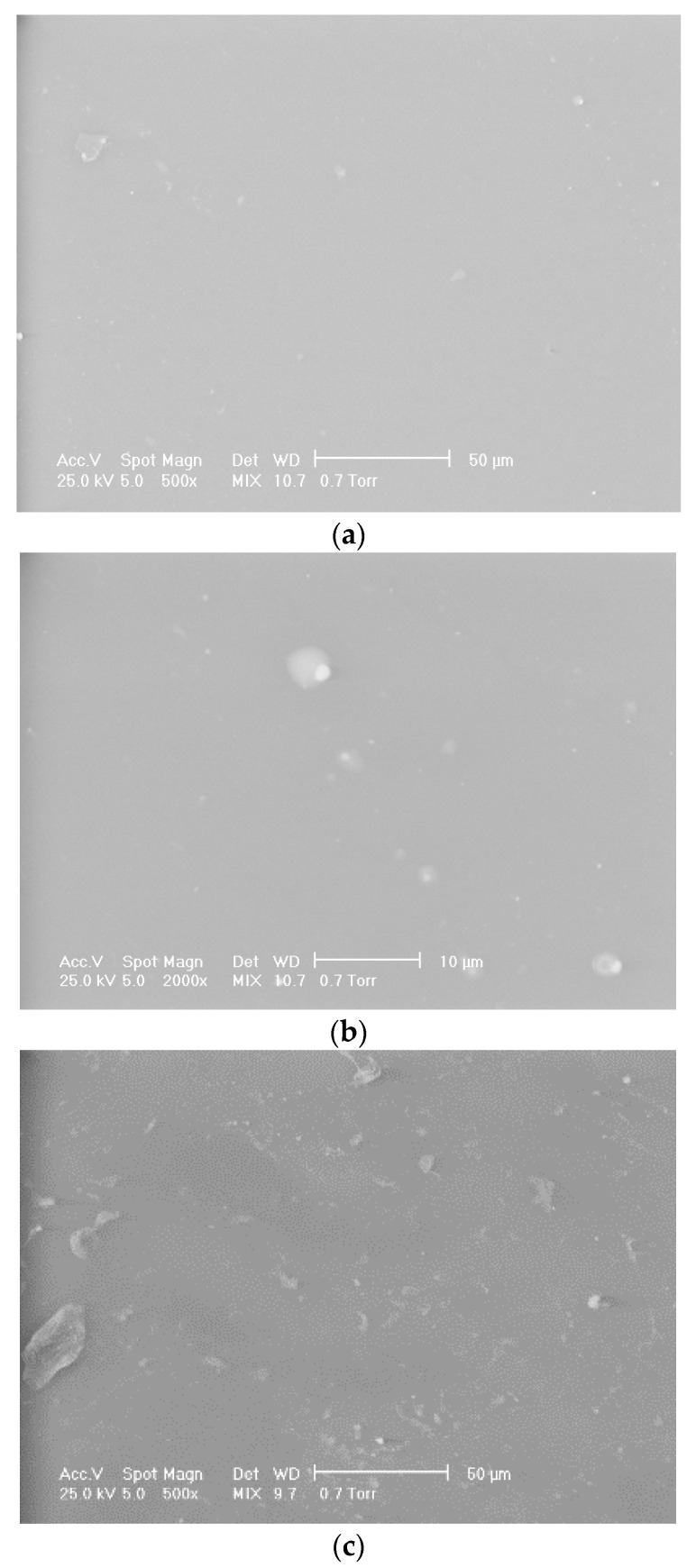

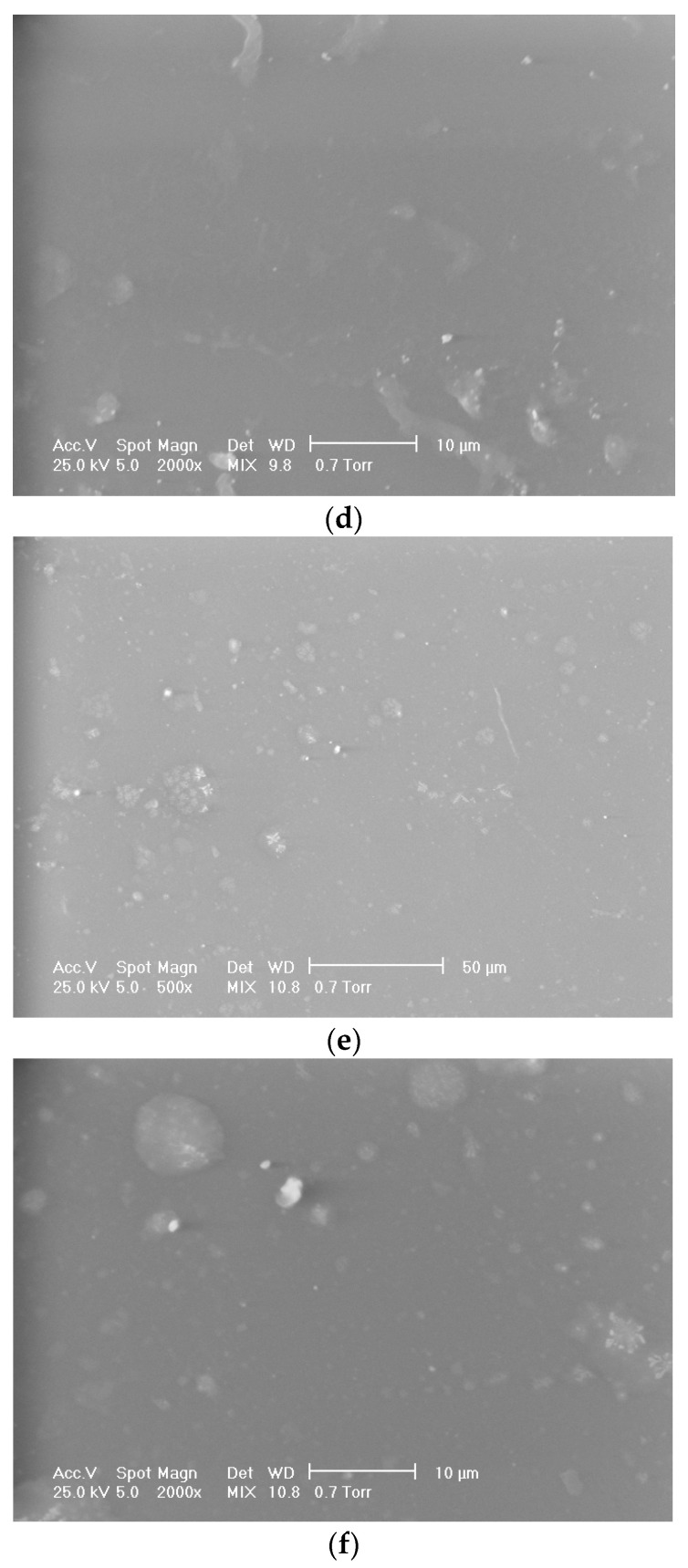
SEM microphotographs of the samples ED0 ((**a**) magnification 500×; (**b**) magnification 2000×), ED1 ((**c**) magnification 500×; (**d**) magnification 2000×), and ED2 ((**e**) magnification 500×; (**f**) magnification 2000×).

The scanning electron microscopy (SEM) images of the control sample ED0 indicated a homogeneous structure, although traces of impurities inside the material can be observed in Figure 4a,b. In addition, smooth surfaces appeared for all samples. A different microstructure can be seen in samples subjected to surface treatment by functionalization in plasma and magnetron sputtering physical evaporation. In Figure 4c,d, which show micrographs with magnifications of 500× and 2000×, respectively, it can be observed that the morphology of the ED1 sample changed. Globular formations consisting of zones with elongated and irregular shapes and different dimensions appeared on the material surface and in its immediate vicinity. These formations have dimensions between 75 and 320 nm, and their dispersion creates the impression of a lusterless material. The impact of the magnetron sputtering treatment can be seen in Figure 4e,f. In this case, the globular formations increased their size to approximately 120–570 nm, and, in some areas, they have an extended elongated character. A similar behavior was reported in the case of the plasma modification of PVC material by Janik et al. [63], Belinger et al. [64], Khorasani et al. [65], and Pedrosa et al. [66]. In addition, a study of PTFE deposition on surfaces, using PED (pulsed electron beam) and PLD (coatings deposited by pulsed laser) techniques, showed spherical particulates for PED coatings and a more complex morphology with numerous particulates for PLD coatings [67].

**Figure 5:**
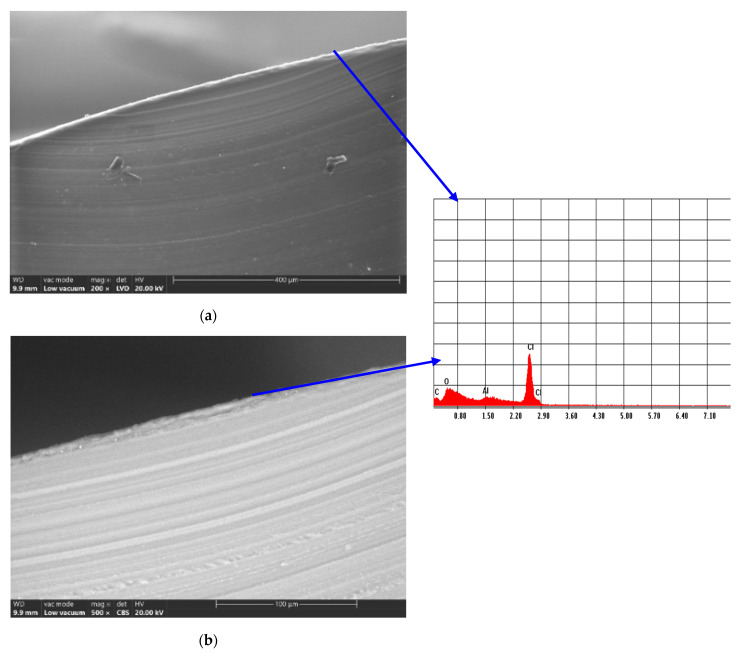

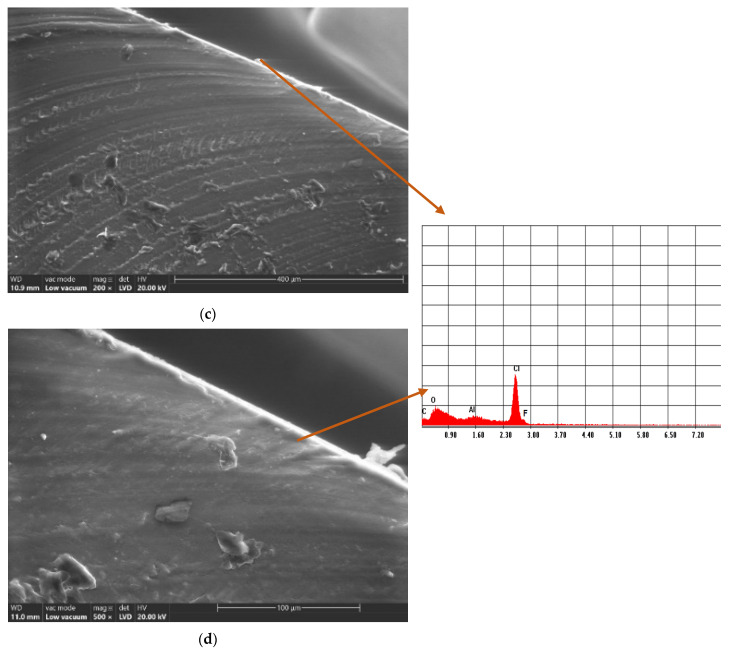

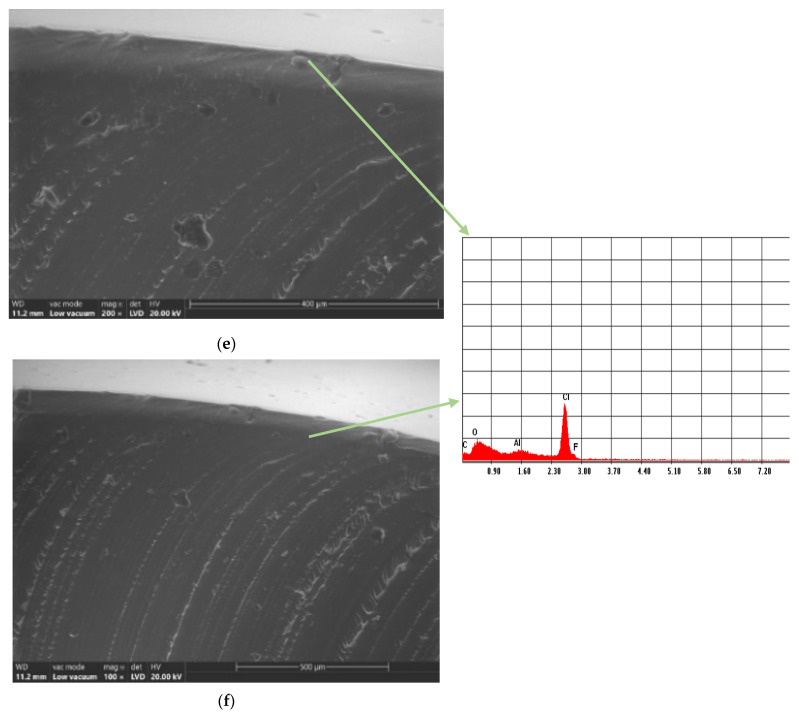
SEM microphotographs in the cross-section of the samples ED0 ((**a**) magnification 200×; (**b**) magnification 500×), ED1 ((**c**) magnification 200×; (**d**) magnification 500×), and ED2 ((**e**) magnification 200×; (**f**) magnification 500×) and the corresponding EDAX spectra.

Figure 5 presents SEM microphotographs in a cross-section of samples ED0, ED1, and ED2 and the corresponding EDAX spectra. In the case of ED1 and ED2, one can observe the outer coating with PTFE. EDAX spectra show the composition of the sample materials. After the plasma treatment, a small peak appears in the EDAX spectra, attributed to the F element, which confirms the presence of the PTFE coating.

#### 3.2.2. Contact Angle

The literature revealed that the contact angle of non-treated PVC is about 75° [68]. Mrad et al. [69] reported a contact angle of 72° in the case of untreated PVC, and, after a low-pressure SF6 plasma treatment was applied, an increase in the contact angle at 93° ± 1 was noticed. It was concluded that the treatment produced a more hydrophobic surface. This phenomenon occurred because the C-H or C-O bonds in the PVC chemical structure were broken, leading to C-F bond formation due to the fluoride (F) ions in the SF6 plasma [55]. The authors observed that the surface changes were permanent, and the fluorination treatment can be considered irreversible even 210 days after it was performed. Similar results for polymeric materials were presented by Resnik et al. [40], who observed surface hydrophobization in the case of SF6 and CF4 plasma treatments. They stated that the dissociation phenomenon of CF4 and SF6 gases produced a high concentration of fluorine ions inside the RF-induced plasma, and, as a result, the saturation of the polymer surface with F functional groups occurred. This effect was much stronger for SF6 than for CF4 due to its higher electronegativity. Babukutty et al. [68] modified a PVC surface with tetrafluoroethylene (PTFE) deposited in an atmospheric pressure glow discharge medium. They developed a PTFE-like coating on the PVC substrate, and a variation in the contact angle between 90° and 103° was reported. As a general conclusion, adding a fluorine layer onto the surface produces an increase in the contact angle and a more hydrophobic character of the surface, a fact that can be considered beneficial against biofilm apparition.

**Figure 6:**
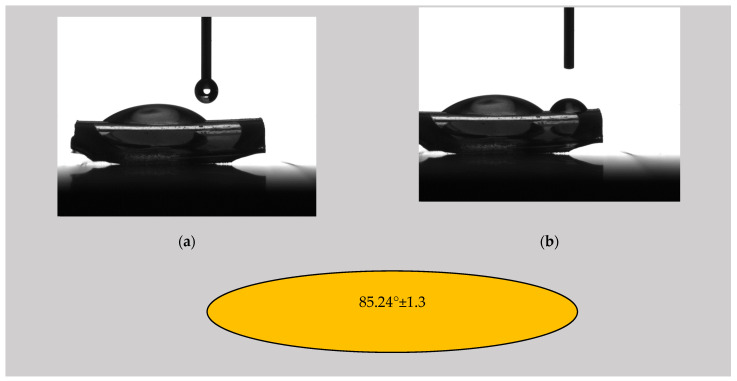
(**a**,**b**) Photographic image of the determination of the contact angle by the sessile drop method for the control sample ED0; contact angle value for the control sample ED0 using distilled water as the wetting agent, respectively.

**Figure 7:**
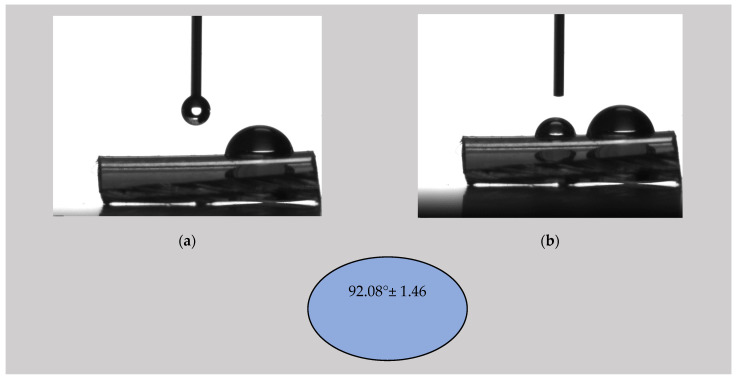
(**a**,**b**) Photographic image of the determination of the contact angle by the sessile drop method for the control sample ED1; contact angle value for the control sample ED1 using distilled water as the wetting agent, respectively.

**Figure 8:**
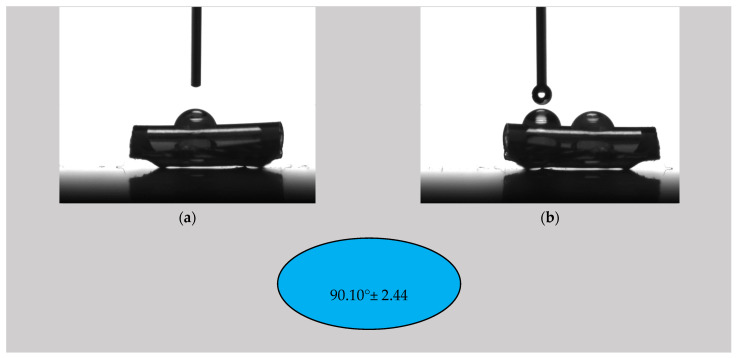
(**a**,**b**) Photographic image of the determination of the contact angle by the sessile drop method for the control sample ED2; contact angle value for the sample ED2 using distilled water as the wetting agent, respectively.

Figure 6, Figure 7 and Figure 8 show images captured during the determination of the contact angle values in the analyzed samples and the contact angle values of the ED0, ED1, and ED2 samples as suggestive shapes.

Water is the most important liquid for biological wettability investigations because it can be found inside the human body. The other two wetting agents, EG and DIM, were only used to apply the OWKR procedure and compute the surface free energy (SFE). It is well known that water and EG are polar liquids since DIM is considered a non-polar/dispersive liquid. In addition, the SFE component values for each wetting agent are provided in [36].

**Figure 9:**
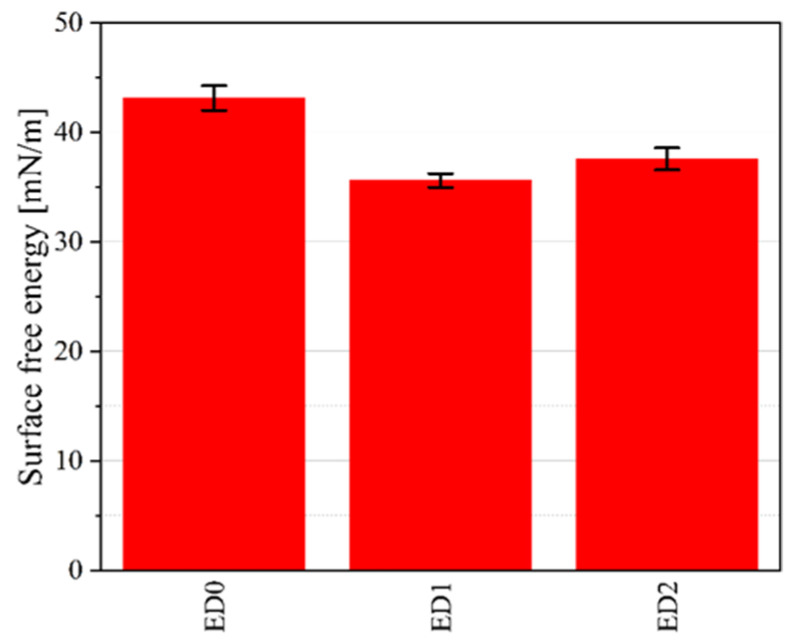
Surface free energy (SFE) results computed based on the OWKR method for the three investigated samples.

The highest contact angle value was obtained for the ED1 sample (92.08° ± 1.46), followed by the ED2 probe (90.10° ± 2.44) and the control sample (85.24° ± 1.30). The control sample exhibited a value close to those found in the literature. For the SFE calculation, we applied the OWKR method [36] and computed the polar and dispersive components of the material’s SFE in the case of the three samples. The average values and standard deviations were chosen for each case, and the total SFE was considered the sum of dispersive and polar interactions at the solid–liquid interface (Figure 9).

It can be observed that the lowest values of SFE correspond to the highest CA, and it can be stated that there is a reduced chance of pathogen adhesion and biofilm formation in the case of the treated samples.

#### 3.2.3. Profilometry Analysis

Profilometry analysis was conducted to investigate the surface topography. The low-inertia stylus profilometer is an adequate device for polymer surface investigations because it does not cause damage to the surface. The results of our analysis show that the control sample exhibited the lowest values for the parameters R_a_ and R_q_ (R_a_ = 0.021 μm; R_q_ = 0.04 μm), followed by the ED2 sample (R_a_ = 0.039 μm; R_q_ = 0.062 μm) and the ED1 sample (R_a_ = 0.056 μm; R_q_ = 0.160 μm) with the highest values. Similar results were reported by Babukutty et al. [69], who found that the untreated PVC surface is flatter and considerably less rough compared to that of the PTFE-coated sample, which exhibited a very fine structure, as is characteristic of PTFE films. The authors reported a 0.07 μm value for the Ra parameter in the case of the coated material. Gengenbach et al. [70,71,72] observed, based on atomic force microscopy (AFM) investigations, the formation of fine additional structures on the plasma-treated surfaces; this can significantly increase the roughness parameter.

**Figure 10:**
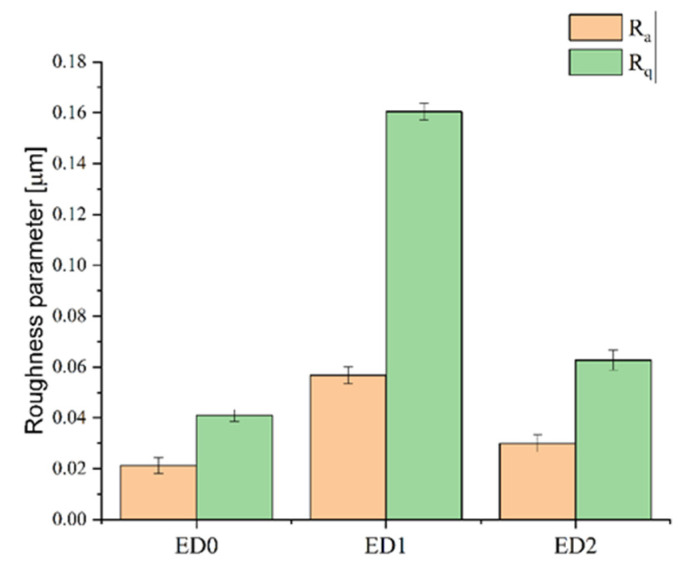
Roughness parameters measured for the three investigated samples.

Figure 10 shows the average values and standard deviations for the roughness parameters determined for the three investigated samples.

It can be observed that the contact angle follows the same trend as the surface roughness due to the F ions’ presence.

### 3.3. Thermogravimetric Analysis (TGA) and Differential Scanning Calorimetry (DSC)

Medical-grade PVC, as a commercial material, exhibits a good thermal stability. The glass transition temperature and melting point values depend on the polymerization temperature, material structure, processing technology, and sample preparation requirements and conditions. Some studies revealed that the macromolecular chain of PVC is mainly syndiotactic and less crystalline [56,73,74]. In addition, the melting point and glass transition values increase at low polymerization temperatures. Other parameters that must be taken into consideration are the molecular weight of the polymer, processing parameters, content, type of additives, and plasticizing effect [75].

**Figure 11:**
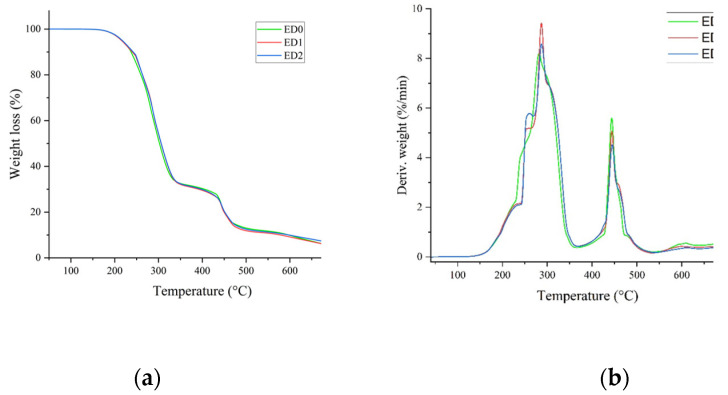
(**a**) TGA and (**b**) DSC thermogravimetric curves for the ED0, ED1, and ED2 samples.

The thermogravimetric curves resulting from the TGA and DSC measurements performed on the control sample (ED0) and surface-treated samples (ED1 and ED2) are presented in Figure 11.

Based on the thermogravimetric analysis, we determined the following parameters: the onset decomposition temperature (T_on_ (°C)), the maximum decomposition rate (T_max_ (°C)), the glass transition temperature (T_g_ (°C)), the residue at 750 °C (R750 (%)), the specific heat capacity (ΔCp (J/(g °C))), and the transition enthalpy (ΔHm (J/g)). Table 2 presents the values of the aforementioned quantities obtained from the experimental measurements.

According to the results obtained from the DSC-TGA experiments (Table 2) and other studies performed on plasticized PVC [76], we can conclude that the values of T_g_ are situated in the vicinity of the −20 °C, which reveals a high content (of around 45–55%) of plasticizer-type Di-ethyl hexyl phthalate (DEHP) in the polymeric recipe, leading to an α-transition in the structure. Since the composition of the ED1 and ED2 samples is similar to that of the control sample, ED0, an increase in the free volume in their structure and an implicit decrease in T_g_ can only be due to surface fluorination from the plasma treatment in SF6 discharge. The values of T_g_ are also related to the density of the samples, but the differences between T_g_ values are small and insignificant. The T_on_ and T_max_ parameters do not exhibit a significant decrease. This fact reveals the good thermal stability of all the samples, since the residue values determined at 750 °C indicate a minimal decomposition of the recipes on the surface of the probes.

### 3.4. Mass Density and Hardness Shore A Measurements

The mass density and hardness Shore A values for the control and surface-treated samples are presented in Table 3.

The measured density values indicate the characteristics of a material plasticized with a high quantity of plasticizing agent. According to Kendall et al. [75], the content of plasticizer can be considered to be about 45–55% wt. This material acts as a lubricant through the polymer chains. Furthermore, increasing the free volume in the polymer allows for much more freedom of movement for the polymeric chains [77].

Regarding the hardness Shore A, the experimental values indicate a plastic and flexible material, able to be processed by extrusion and to support the modification of the shape to obtain a curved tube, as found by Gama et al. [77].

### 3.5. Antimicrobial and Cytotoxicity Analyses

#### 3.5.1. Antimicrobial Tests

Antimicrobial tests were analyzed based on the one-way ANOVA with repeated measures test. All statistical analyses were performed using GraphPad Prism Software, v. 5.03 428 (GraphPad Software, La Jolla, CA, USA, www.graphpad.com, accessed on 20 May 2020). Significant differences were noted as: *p* < 0.05; ** *p* < 0.001; *** *p* = 0.0001. All parameters were evaluated in triplicate, and the results were expressed as the mean and standard deviation (SD) values of three independent determinations. The statistical analysis and average/SD values were calculated using GraphPad Prism^®^ version 9.1.0 (GraphPad Software, La Jolla, CA, USA). A two-way ANOVA followed by a Dunnett’s test was used with a *p*-value of 0.05.

**Figure 12:**
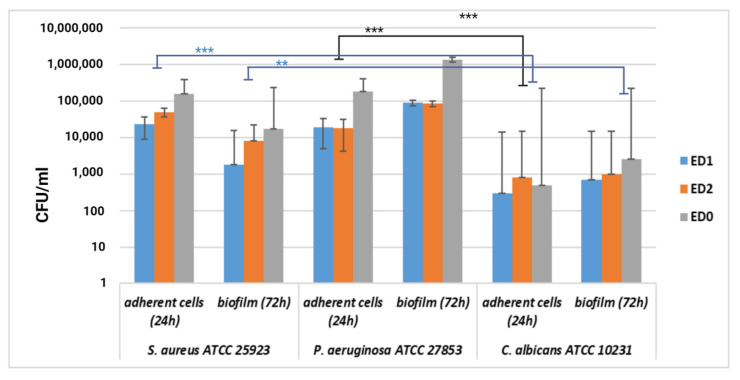
Graphic representation of the log CFU/mL for the comparative evaluation of the anti-adherence and antibiofilm activity of the tested samples. The results were compared using two-way ANOVA and Dunnett’s multiple comparisons tests; *p* < 0.05; ** *p* < 0.001; *** *p* = 0.0001.

The experimental contamination of the tested samples with the standard microbial strains allowed for the quantitative evaluation of their resistance to adhesion and monospecific biofilm development by the determination of CFU/mL values. The values were grouped and analyzed statistically, as shown in Figure 12. It can be observed that significant differences between the adhesion capacity and biofilm development appeared between the bacterial strains and the yeast strain because the bacterial strains were much more adherent to all three types of tested samples. At 72 h, the highest degree of biofilm formation was recorded for the Gram-negative strain *Pseudomonas aeruginosa* (ATCC 27853), with no significant differences regarding the sample type. In general, many medical devices, such as endotracheal tubes, are made from hydrophobic materials, such as PVC, and *Pseudomonas aeruginosa* and *Staphyloccocus aureus* are opportunistic bacteria that cause a wide range of hospital-acquired infections through the contamination of medical devices and biofilm generation [78,79]. Generally, the adherence of cells and biofilm formation on a material surface is governed by hydrophobic interactions, steric interactions, protein adhesion, electrostatic interactions, and Van der Waal forces [80,81]. In our study, we coated the plasticized PVC surfaces with a layer of PTFE, resulting in superhydrophobic surfaces, which correlates with a reduced adhesion capacity of the yeast cells but not of the bacterial cells, perhaps because the cell wall structure is different in yeasts. On the other hand, both *P. aeruginosa* and *S. aureus* are bacteria species possessing outer membrane proteins and lipopolysaccharides that provide surface charges that moderate cell adhesion [82]. Thus, the mechanisms are too dependent on environmental conditions to establish with certainty which of the factors influences the ability to form biofilms the most.

The least adherent strain, after 24 h of incubation, was *Candida albicans* (ATCC 10231). *C. albicans* can exist in three phases: budding yeast, pseudohyphae, and hyphae. Budding yeast has a unicellular morphology and can be involved in biofilm formation, being found in in vitro cultures in the log phase of the cell cycle after 24 h of incubation [83,84]. *Candida albicans* is also an opportunistic microorganism with two distinct morphotypes: round-to-ovoid-shaped yeast cells and filamentous hyphae [85]. The hyphal phase, developed after 48 h of incubation, is responsive to stabilized mature biofilms, but only in in vivo conditions, not in vitro [85]. This also may explain the significantly lower CFU/mL values obtained in our experiment compared with the bacterial strains’ results.

#### 3.5.2. Cytotoxicity Analysis

**Figure 13:**
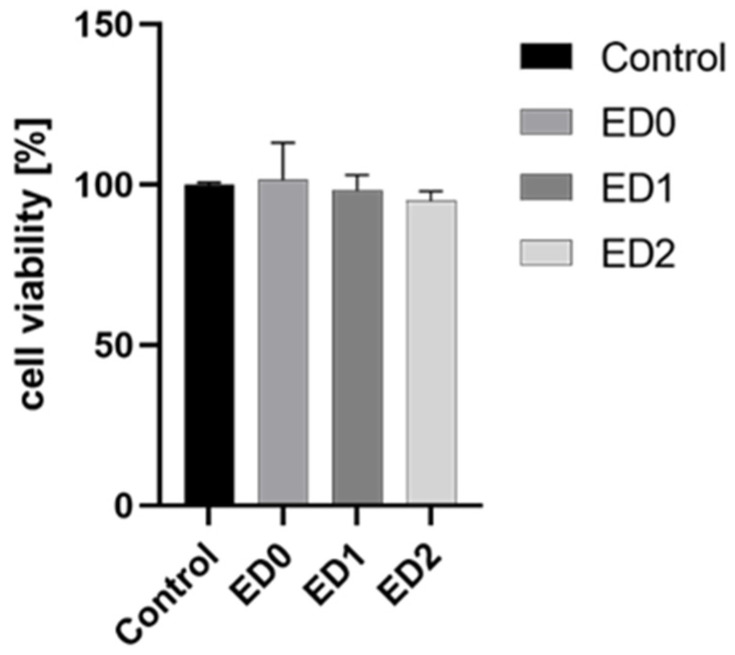
Graphic representing the cytocompatibility test results; there were no statistically significant differences between the samples and the control.

All tested samples showed a high compatibility with the HT-29 human cell line, with no statistically significant differences between the samples and the control (Figure 13).

## 4. Conclusions

In recent years, due to the increase in the number of lung diseases and, especially, in the context of the coronavirus pandemic from 2019 to 2022, worldwide demands for endotracheal-tube medical devices have increased. In hospital units and, especially, in intensive care units, the intubation of patients with endotracheal tubes led, to a large extent, to the development of nosocomial superinfections or coinfections after a period of use of several days. According to the presented biological studies, the PVC material favors the formation of a biofilm on the surface of the endotracheal tube after 48 h of intubation.

Due to these facts, in order to remove most of the inconveniences related to the material with which ETTs are produced, we produced, in our laboratory research, two methods of improving the wettability by increasing the hydrophobic character in order to eliminate the growth of biofilms on ETT surfaces.

The results obtained from the testing of the experimental samples reveal the success of using PTFE magnetron sputtering deposition combined with the SF6 gas: the hydrophobic character increased, glossy surfaces without roughness were obtained, and the antimicrobial and biocompatibility effects of the surfaces were observed.

These results direct us to continue research in this field, namely, to move towards the optimization of the irradiation process in order to obtain ETTs with a longer duration of use.

## Figures and Tables

**Table 1:** Atomic composition of the investigated ED0, ED1, and ED2 materials.

Element	Atomic Composition (%)		
	ED0	ED1	ED2
C1s	77.8	58.9	69.8
O1s	13.7	12.9	11.4
Cl2p	5.4	0.3	-
Si2p	2.8	2.7	-
F1s	-	23.1	11.9
N1s	-	1.7	-
I3d	-	-	3.0
Zn2p3	-	-	3.7

**Table 2:** TGA and DSC parameters obtained for the investigated samples.

	Glass Transition			Relaxation Enthalpy			Melting Enthalpy			R_750_	
	T_on_	T_g_	ΔC_p_	T_on_	T_max_	ΔH_m_	T_on_	T_max_	ΔH_m_	N_2_	Air
	°C	°C	J/(g °C)	°C	°C	J/g	°C	°C	J/g	%	%
ED0, 1st heating	−35.9	−15.1	0.25	–	–	–	48.4	66.2	1.81	1.85	0.01
ED0, cooling	7.8	−15.0	0.31	-	-	-	-	-	-		
ED0, 2nd heating	−33.7	−6.7	0.32	-	-	-	-	-	-		
ED1, 1st heating	−38.8	−19.9	0.25	116.1	121.8	0.57	50.4	67.4	1.73	2.41	0.01
ED1, cooling	9.8	−9.3	0.34	79.0	73.8	0.61	-	-	-		
ED1, 2nd heating	−36.0	−13.0	0.31	-	-	-	113.4	120.4	0.70		
ED2, 1st heating	−39.1	−19.2	0.24	115.9	121.7	0.58	51.3	67.5	1.93	3.98	0.02
ED2, cooling	8.1	−20.8	0.33	79.0	73.8	0.59					
ED2, 2nd heating	−35.7	−11.3	0.30	-	-	-	113.4	120.4	0.73		

**Table 3:** The mass density and hardness Shore A experimental values.

Sample	Density (g/cm^3^)	Hardness Shore (°Sh A)
ED0	1.23	83
ED1	1.21	81
ED2	1.21	79

## Data Availability

Not applicable.
